# Determination of silicon dioxide in fluorite by ICP OES with closed digestion and boric acid complex reaction

**DOI:** 10.1371/journal.pone.0338898

**Published:** 2025-12-11

**Authors:** Xiao Wang, Liming Gan, Jiufen Liu, Xin Wei, Xi Wang, Tao He, Na Guo

**Affiliations:** 1 Xi’an Mineral Resources Survey, China Geological Survey, Xi’an, China; 2 Technology Innovation Center for Gold Ore Exploration, China Geological Survey, Xi’an, China; 3 Natural Resources Survey, China Geological Survey, Beijing, China; Hamadan University of Medical Sciences, IRAN, ISLAMIC REPUBLIC OF

## Abstract

Fluorite is widely utilized in the new energy, semiconductor, high-end manufacturing, and steelmaking industries. The silicon dioxide (SiO₂) content is critical for both the quality evaluation of fluorite and the design of beneficiation processes. However, the traditional fusion method suffered from several limitations, including cumbersome pretreatment, silica precipitation during acidification, and significant matrix effects caused by alkali metals introduced during fusion. To address these challenges, this study developed an innovative method employing acid dissolution for the determination of SiO₂ content in fluorite using inductively coupled plasma optical emission spectrometry (ICP OES). This method employs a mixture of nitric acid and hydrofluoric acid in a water bath to digest the sample within a closed system. Systematic optimization experiments were conducted to evaluate key parameters, including the volumes of nitric acid and hydrofluoric acid, digestion time in the water bath, and the quantity of boric acid solution, with the aim of enhancing analytical performance. Complete dissolution of silicon dioxide in fluorite was achieved by treating 0.2000 g of sample with 5.0 mL nitric acid and 2.0 mL hydrofluoric acid, followed by heating at 30 minutes. Subsequently, excess fluoride ions were complexed by adding 10 mL of a 50 mg mL^-1^ boric acid solution. After dilution, the samples are analyzed by ICP OES. At a dilution factor of 2500, the limit of detection (LOD) for SiO₂ was 0.974 µg g^-1^, with a measurement range spanning from 0.0004% to 25%. Method validation was performed by analyzing six fluorite certified reference materials in seven replicates. The results were in good agreement with certified values, with relative errors (RE) ≤ 3.61%. Seven replicate analyses of five real fluorite samples also showed consistency with results obtained using the standard alkaline fusion colorimetric method (GB/T 5159.8–2006), with relative deviations ≤ 2.71% and relative standard deviations (RSD, n = 7) ≤ 3.04%. This method employs nitric acid and hydrofluoric acid under sealed water bath digestion conditions for the dissolution of fluorite samples, effectively preventing the volatilization loss of silicon tetrafluoride formed during the reaction between fluorine and silicon. By introducing boric acid to complex excess fluoride ions, the interference caused by residual hydrofluoric acid is eliminated, thereby enabling the use of conventional sampling systems that are otherwise incompatible with hydrofluoric acid media. This approach facilitates the rapid determination of silicon dioxide in fluorite via acid dissolution combined with ICP OES analysis. The procedure is straightforward, efficient, and cost-effective, significantly enhancing analytical throughput. It is well suited for the routine and high-throughput determination of SiO₂ in large batches of fluorite samples.

## Introduction

Fluorite, also known as fluorspar, is a non-metallic mineral predominantly composed of calcium fluoride (CaF₂) [1–3]. Given its scarcity and non-renewable nature, it is considered a strategic mineral resource by countries such as the United States, the European Union, China, Japan, and Iran [4–6]. As a vital industrial raw material, fluorite finds extensive application in key sectors including new energy, semiconductors, high-end manufacturing, and steelmaking [7–9]. In addition to calcium fluoride, fluorite often contains impurities such as silicon dioxide (SiO₂), calcium carbonate (CaCO₃), magnesium carbonate (MgCO₃), phosphorus (P), sulfur (S), arsenic (As), and iron (Fe) [10]. Among these impurities, SiO₂ is the most significant. Its mass fraction is a critical parameter for assessing the grade of fluorite concentrate and serves as an essential reference for optimizing beneficiation processes. The accurate and rapid determination of SiO₂ content in fluorite holds great importance for guiding mineral resource exploration and development [11–14].

The determination of SiO₂ content in fluorite currently employs several methods, including gravimetric analysis [15,16], spectrophotometry [17–21], X-ray fluorescence spectrometry (XRF) [22–24], neutron activation analysis [25], Raman spectroscopy [26], inductively coupled plasma mass spectrometry (ICP-MS) [27], and inductively coupled plasma optical emission spectrometry (ICP OES) [28–31]. Among these techniques, gravimetric analysis and spectrophotometry are commonly used for SiO₂ determination in fluorite. However, these methods involve labor-intensive sample pretreatment and are prone to matrix interference. XRF, although simple to operate, is not suitable for determining low SiO₂ content. In contrast, ICP OES offers advantages, such as rapid analysis, a wide linear range, low detection limits, and high accuracy, making it widely adopted [32]. Regarding sample preparation for SiO₂ determination in fluorite using ICP OES, common methods include alkaline fusion and acid digestion. For example, Pu et al. [33] employed a lithium tetraborate-lithium metaborate composite flux for sample fusion, followed by acidification and ICP OES determination of silicon and other elements in fluorite. However, this alkaline fusion method introduces a significant amount of salts, leading to pronounced matrix interference during testing. Nian et al. [34] utilized a closed microwave digestion system with a mixture of hydrochloric acid, nitric acid, and hydrofluoric acid for sample digestion, combined with a custom-built hydrogen fluoride-resistant ICP OES system for the determination of silicon and other elements in fluorite. While effective, the use of a hydrogen fluoride-resistant system increases costs and limits its widespread application.

Based on previous studies, this study employs a closed nitric acid-hydrofluoric acid (HNO₃-HF) water bath digestion method to treat fluorite samples, preventing the reaction between fluorine and silicon that produces volatile silicon tetrafluoride (SiF_4_), thereby minimizing analyte loss [29,35]. To effectively eliminate fluoride ions, excess boric acid is added to form stable fluoro-borate complexes. Boric acid is also used for matrix matching in the calibration standards. A conventional quartz-based sampling system is utilized, with boric acid serving as the primary medium for determination by ICP OES. Instrumental memory effects from silicon are mitigated by rinsing with a dilute ammonia solution. This work establishes a novel acid dissolution-based ICP OES method for the determination of SiO_2_ in fluorite. The method enables silica decomposition through acid digestion and overcomes the challenge of quartz component corrosion by hydrofluoric acid, which previously hindered direct ICP OES analysis. However, a limitation remains: due to the lack of high-purity SiO_2_ standard reference materials in fluorite matrices, further investigation into the accurate quantification of high-silica samples (SiO_2_ ≥ 25%) has not been conducted.

## Materials and methods

### Instruments

The instrument parameters for the determination of silicon were established in accordance with the national standard method “Inductively Coupled Plasma Optical Emission Spectrometry Method for the Determination of Silicon, Aluminum, Iron, Potassium, Magnesium, and Titanium in Silica” (GB/T 5159.16-2017). Combined with the recommended parameters of the iCAP-PRO type ICP OES (PerkinElmer Company, USA), the optimized operating parameters for ICP OES were selected (Table 1). The analytical spectral line for silicon was chosen as 251.611 nm, and the direct observation mode was adopted, which enables accurate determination of silica.

**Table 1:** Optimization parameters of instrument analysis experiment.

Item	Parameter	Item	Parameter
Power of RF	1150 W	Test number	3
Speed of flush pump	75 rpm	Washing time	25 s
Gas flow of auxiliary	0.5 L min^-1^	Atomizer	Salt-tolerant
Gas flow of atomizer	0.60 L min^-1^	Spectral lines of silicon	251.611nm

The analytical balance (BS124S, Sartorius, Beijing) with a sensitivity of 0.01 mg was used. The ultrapure water system (MILLI-Q ADVANTRGE A10) with a resistivity of 18.25 MΩ·cm was used for sample preparation.

### Reagents and solutions

The chemicals, including nitric acid, hydrofluoric acid, and boric acid, were obtained from Chengdu Kelon Chemical Co., Ltd. (Chengdu, China). A standard solution of silicon dioxide (SiO_2_) was purchased from the National Analytical and Testing Center for Non-ferrous Metals and Electronic Materials, comprising 1000 µg mL^-1^ SiO_2_ in 2.0 mol L^-1^ hydrofluoric acid.

The matrix matching of standard solution was employed to minimize interference from the borate medium in silica determination. Specifically, 10 mL of a 50 mg mL^-1^ borate solution was added to each standard solution, ensuring consistent borate levels across standard reference materials and sample digestion procedures.

For the silica standard series, appropriate volumes of the silica stock standard solution were accurately pipetted, and 10 mL of a 50 mg mL^-1^ borate solution was added. The mixtures were then diluted to 100 mL with a 2% (v/v) hydrofluoric–nitric acid mixture, yielding silica concentrations of 0, 1.0, 2.0, 5.0, 10, 20, 50 and 100 µg mL^-1^.

### Samples

Actual samples of fluorite: In May 2025, five fluorite ore samples (designated XS1-XS5)-representing lean, associated, and rich ore types-were collected from a delineated mineralized zone in the Xiaobaihe Gully and Jianghe District, Ruoqiang County, Baoyingguan Mongolian Autonomous Prefecture, Xinjiang Uygur Autonomous Region, China. After transport to the laboratory, samples were air-dried, crushed, ground, and sieved through a 200-mesh sieve. They were then stored in light-protected paper bags within a controlled storage room (temperature: 20–25°C; relative humidity: 20–60%; light protection) pending method comparison and validation in July 2025.

Fluorite reference material: The reference materials coded GBW07250, GBW07251, GBW07253, and GBW07254 were developed by the Technology Center of Wuhan Iron and Steel (Group) Co., Ltd.; those coded GBW(E) 070102 and GBW(E) 070106 were supplied by the Jinan Quandong Standard Material Research Institute. The standard material was stored in compliance with the certificate-specified conditions, including placement in a designated storage cabinet under controlled environment (temperature: 20–25°C; relative humidity: 20–60%; light protection). Single-factor optimization and method validation experiments were conducted in June 2025.

### Experimental procedure

**Fig 1:**
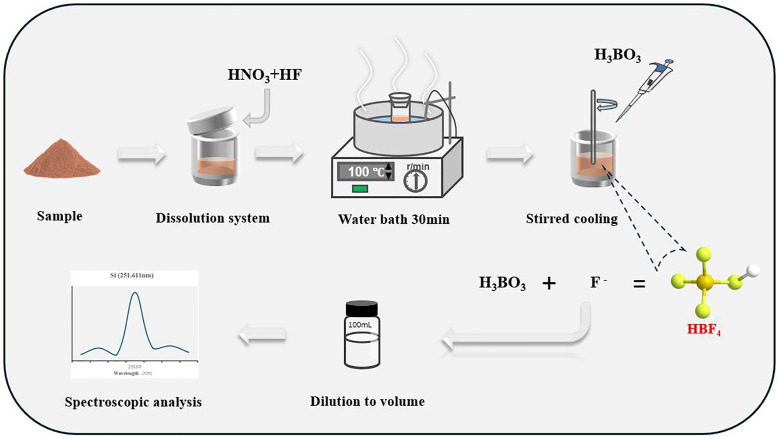
The flowchart of the experiment.

Fluorite samples were digested under closed conditions using a mixture of nitric acid and hydrofluoric acid. Excess boric acid was added to facilitate fluoride ion complexation in the solution. Following dilution to the desired volume, the samples were analyzed by inductively coupled plasma optical emission spectrometry (ICP OES). The experimental workflow is depicted in Fig 1.

Three fluorite reference materials (GBW07251, GBW07253, and GBW07254), with silicon contents ranging from 0.87% to 14.15%, were employed in a univariate experimental design to optimize the analytical method. The nitric acid volume was optimized within the range of 2.0–7.0 mL, hydrofluoric acid within 0.5–3.0 mL, water bath digestion time within 15−40 minutes, and borate solution volume within 4.0–12.0 mL, to evaluate their effects on analytical performance. The determination parameters for the ICP OES instrument were established based on the standard “Fluorspar-Determination of Silicon, Aluminum, Iron, Potassium, Magnesium, and Titanium Content by Inductively Coupled Plasma Mass Spectrometry” (GB/T 5159.16−2017) and the manufacturer’s recommended operating conditions, leading to the identification of optimal experimental conditions for the determination of silicon dioxide.

### Method validation

The method was validated in accordance with the recommended Chinese National Standard (GB/T 27417−2017) method-Guidelines for Confirmation and Validation of Chemical Analysis Methods. The blank standard deviation method assesses LOD by analyzing multiple blank samples or blanks spiked with the lowest acceptable concentration. In this article, blank values were determined using 11 low-concentration samples, and the corresponding standard deviation (S) was calculated. Limit of Detection (LOD) is determined according to Equation (1). Limit of Quantification (LOQ) was defined as three times the LOD. The measurement range of the method was subsequently defined based on these values. The analytical measurement range was thereby established.


LOD=0+3S (1)

To assess the precision and accuracy of the method, six certified fluorite reference materials (GBW 07250, GBW 07251, GBW 07253, GBW 07254, GBW(E) 070102, GBW(E) 070104) and actual fluorite samples were analyzed in parallel. The SiO₂ content in the reference materials ranged from 0.84% to 18.11%, and from 0.56% to 14.59% in the actual samples. Comparative analysis across these samples was conducted to evaluate method performance.

## Results and discussion

### The impact of nitric acid amount on silicon dioxide content

Fluorite often contains impurities such as calcium carbonate, iron oxide, and aluminum oxide [36,37]. Adding nitric acid can effectively digest calcium carbonate and other impurities in fluorite samples, reducing the loss of hydrogen fluoride during the digestion of SiO₂. According to the Chinese national standard method “Analysis Method of Cathodic Carbonates” (GB/T 10304−2008) [38], 5.0–7.0 mL of nitric acid is required for the dissolution of carbonates. Given that this study employs a closed digestion procedure, which minimizes nitric acid loss due to volatilization, three fluorite certified reference materials were selected and tested across a nitric acid volume range of 2.0–7.0 mL to optimize the acid quantity used in this work.

**Fig 2:**
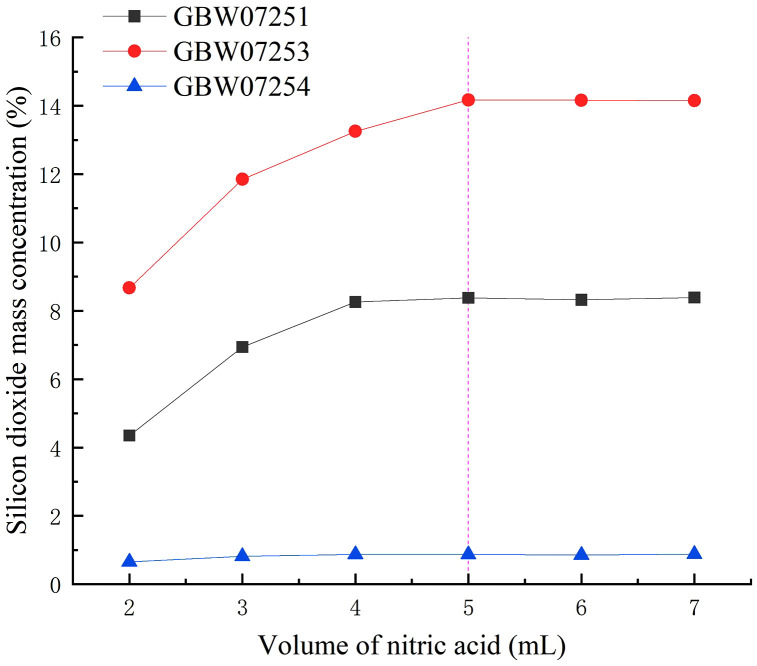
The impact of different volumes of nitric acid on the results of silicon dioxide.

Six replicate samples of fluorite standard reference materials (GBW07251, GBW07253, and GBW07254), each weighing 0.2000 g and containing silicon dioxide in the range of 0.84% to 14.12%, were prepared. Subsequently, nitric acid was added in varying volumes (2.0, 3.0, 4.0, 5.0, 6.0, and 7.0 mL, respectively) to each sample, and the silicon dioxide content was determined following the established experimental procedure. The experimental results (Fig 2) showed that the measured values gradually stabilized with increasing nitric acid volume, when the nitric acid volume reached 5.0 mL, the results for low, medium, and high SiO₂ contents remained stable. Therefore, the optimal nitric acid volume was determined to be 5.0 mL. For more data information, see S1 Fig.

### The impact of hydrofluoric acid dosage on silicon dioxide content

Hydrofluoric acid reacts with silicon dioxide to form silicon tetrafluoride, which is widely used for the digestion of silicate phases in samples. Hydrofluoric acid effectively digests silicon dioxide in fluorite samples. Nian Jiqiang et al. [34] employed 5 mL of hydrofluoric acid to digest silicon, iron, magnesium, potassium, sodium, phosphorus, and sulfur in fluorite. In the present study, only silica was targeted for decomposition, and hydrofluoric acid didn’t undergo volatilization or loss during the closed digestion process. Therefore, the dosage of hydrofluoric acid was optimized using three fluorite certified reference materials, with volumes ranging from 0.5 to 3.0 mL.

**Fig 3:**
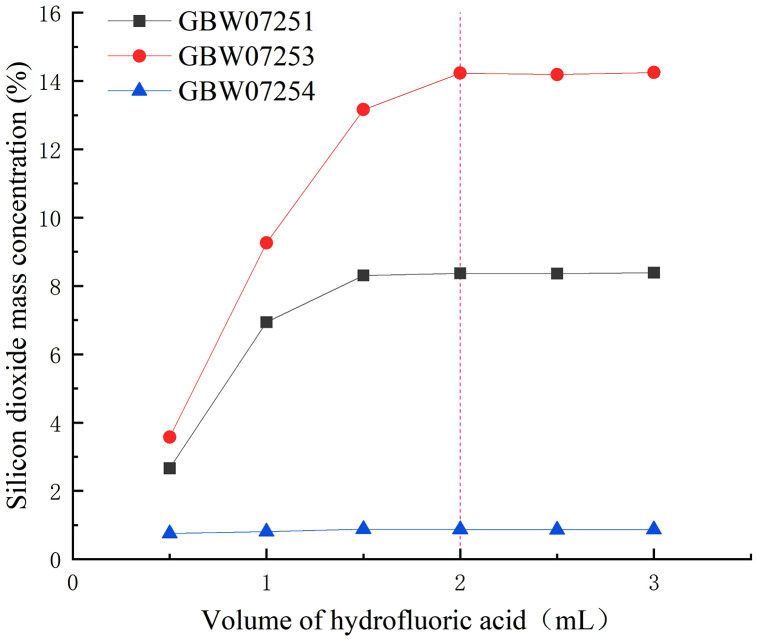
The impact of different volumes of hydrofluoric acid on the results of silicon dioxide.

Experimental results (Fig 3) demonstrated that the measured values gradually stabilized as the hydrofluoric acid volume increased. When the hydrofluoric acid volume exceeded 1.5 mL, the results for low silicon dioxide content remained stable. At volumes exceeding 2.0 mL, the results for high silicon dioxide content stabilized. Therefore, the optimal hydrofluoric acid volume was determined to be 2.0 mL. The optimal dosage of hydrofluoric acid was lower than literature-reported values for digesting silicon, iron, magnesium, potassium, sodium, phosphorus, and sulfur. This indicates that besides SiO₂, the decomposition of iron, magnesium, potassium, and sodium compounds also consumes hydrofluoric acid. For more data information, see S2 Fig.

### The impact of digestion time on silicon dioxide content

The dissolution time of fluorite samples greatly affects the dissolution rate of SiO₂. Increasing the dissolution time improves the completeness of SiO₂ dissolution, thereby enhancing the accuracy of subsequent determinations. Nian Jiqiang et al. [34] employed microwave digestion to decompose silicon, iron, magnesium, potassium, sodium, phosphorus, and sulfur in fluorite, achieving complete dissolution within approximately 25 minutes. In this study, three fluorite standard reference materials were utilized to optimize the water bath digestion time over a range of 15–40 minutes.

**Fig 4:**
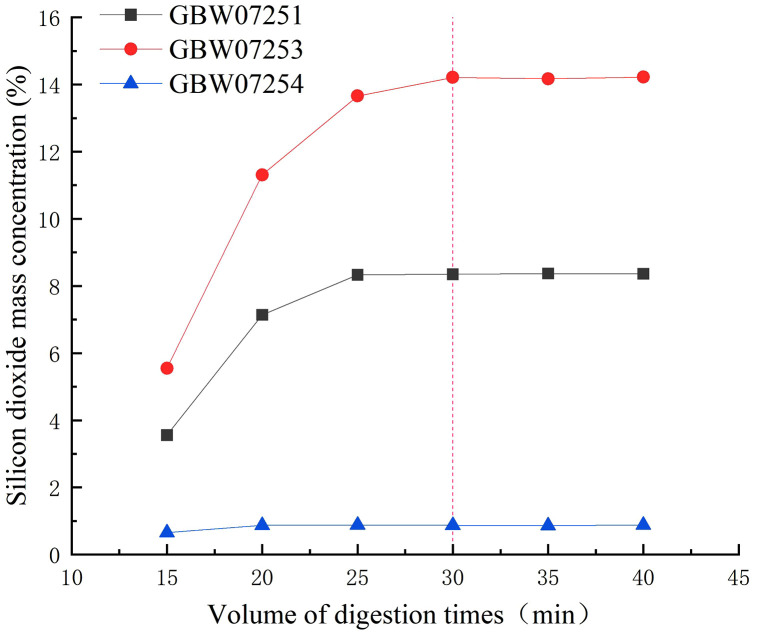
The impact of different digestion times on the results of silicon dioxide.

Six replicate portions of 0.2000 g each of fluorite standard reference materials (GBW07251, GBW07253, and GBW07254) were accurately weighed, followed by the addition of 5 mL of nitric acid and 2 mL of hydrofluoric acid. The samples were subjected to closed-vessel water bath heating digestion for durations of 15, 20, 25, 30, 35, and 40 minutes, respectively. Subsequently, the silicon dioxide content was determined using the established experimental procedure. Experimental results (Fig 4) revealed that the measured values gradually stabilized as the digestion time increased. For low-content SiO₂, the results remained stable when the digestion time exceeded 25 minutes, while for high-content SiO₂, stabilization occurred when the digestion time exceeded 30 minutes. Therefore, the optimal sample dissolution time was determined to be 30 minutes, which is slightly longer than the digestion time reported in the literature for silica under microwave-assisted conditions. This suggests that silica present in fluorite can be relatively easily and completely dissolved. For more data information, see S3 Fig.

### The impact of boric acid dosage on silicon dioxide content

Boric acid reacts with hydrofluoric acid at room temperature to form fluoboric acid, effectively removing residual hydrofluoric acid from the digestion solution [39]. Due to the strong fluorine-boron affinity, fluoboric acid does not dissociate into fluoride ions at room temperature, thereby preventing quartz corrosion and enabling routine ICP OES determination. In the experimental procedure, 2 mL of hydrofluoric acid was introduced, corresponding to an approximate fluoride ion content of 0.0548 mol. Fluoride ions [F]^-^ were complexed with boric acid (H_3_BO_3_) in a stoichiometric ratio of 1:4 to form the [BF4]^-^ complex. Based on this stoichiometry, the theoretical requirement of boric acid was calculated to be 0.0137 mol. To achieve effective complexation, 17 mL of a boric acid solution with a mass concentration of 50 g L^-1^ was added. However, considering that a portion of the hydrofluoric acid reacted with silicon dioxide present in the sample, thereby reducing the amount of free hydrofluoric acid available in solution, the actual boric acid demand was lower than theoretically predicted. To account for this variation and optimize the boric acid addition, three fluorite standard reference materials were employed, and the volume of boric acid solution was adjusted within the range of 4–12 mL.

**Fig 5:**
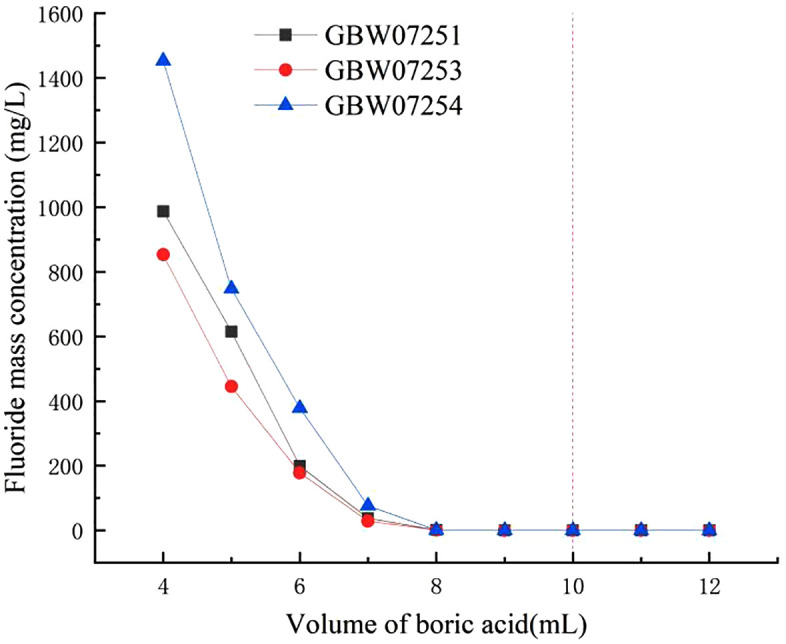
The impact of different volumes of boric acid on the results of fluoride ion.

Six aliquots of each fluorite standard reference material (GBW07251, GBW07253, GBW07254) were accurately weighed to 0.2000 g. Subsequently, 5 mL of nitric acid and 2 mL of hydrofluoric acid were added to each sample, followed by sealed-vessel digestion in a Boiling water bath for 30 minutes. Thereafter, 4.0, 5.0, 6.0, 7.0, 8.0, 9.0, 10.0, 11.0, and 12.0 mL of 50 g L^-1^ boric acid solution were added incrementally to separate samples. Each solution was stirred thoroughly using a plastic rod for 1–2 minutes, cooled to room temperature, and then transferred quantitatively into a 100 mL polyethylene volumetric flask. The final volume was adjusted with deionized water, and the solutions were shaken vigorously to ensure homogeneity. After allowing the solutions to clarify, the mass concentration of fluoride ions was determined by ion chromatography. Experimental results (Fig 5) demonstrated that fluoride ion concentration gradually decreased with increasing boric acid addition. For GBW07251 and GBW07253 samples, negligible fluoride ion concentration was observed when boric acid volume exceeded 8 mL. Similarly, for GBW07254, negligible fluoride ion concentration was achieved when the boric acid volume exceeded 10 mL. Therefore, the optimal volume of boric acid employed was 10 mL. This volume is less than the 17 mL required for complete complexation with 2 mL of hydrofluoric acid, suggesting that a portion of the hydrofluoric acid was consumed during the dissolution of silica. For more data information, see S4 Fig.

### Optimization of measurement conditions

Fluorite samples were dissolved using a mixed acid under water bath heating. After dissolution, boric acid was added to complex fluoride ions, leaving the sample solution primarily containing calcium, silicon, and boron. The selection of analytical lines for silicon was systematic and based on the following criteria: sensitivity, avoidance of spectral interference, non-structural background, sufficient linear range, and practical applicability. Based on these considerations, the analytical line for silicon was selected at Si 251.611 nm.

Fluorite is primarily composed of calcium fluoride. However, CaF₂ has a low dissolution rate in mixed acid, minimizing calcium matrix effects. Boric acid was added to the sample solution to complex excess hydrofluoric acid. Since boric acid can influence silicon determination, matrix-matching techniques using standard solutions were employed to eliminate the effect of the boric acid medium on silicon measurements. In this study, when preparing the standard solution series, 10 mL of boric acid solution (50 mg mL^-1^) was added to achieve matrix matching and ensure accurate determination of silicon in fluorite samples.

### Limit of detection (LOD) and detection range

Eleven blank samples were prepared and analyzed by ICP OES. Eleven blank samples were prepared and analyzed by ICP OES. According to the description in the “Method Validation”, the LOD was calculated as three times the standard deviation of the blanks, and the Limit of Quantification (LOQ) was obtained by multiplying the LOD by 3. It can be concluded that the LOQ for SiO₂ is 3.92 µg g^-1^, with a determination range of 0.0004% to 25% (Table 2), which is adequate to meet the analytical requirements for silicon dioxide determination in fluorite samples.

**Table 2:** LOD and detection ranges of silicon dioxide.

Element	Blank measurement value (%)	Standard deviation (%)	LOD (µg g^-1^)	LOQ (µg g^-1^)	Detection range (%)
SiO_2_	2.23 3.15 2.54 3.16 3.29 3.15 2.92 3.11 3.16 2.72 3.01	0.325	0.974	3.92	0.0004 ~ 25

### Precision and accuracy of the method

Seven parallel determinations were performed on six national standard materials (GBW07250, GBW07251, GBW07253, GBW07254, GBW(E) 070105, and GBW(E) 070106) using the experimental method. The measured results were consistent with the standard values, with RE (Relative Errors) for all elements below 3.61% (Table 3). For more data information, see S1 Table.

**Table 3:** The RSD and RE of this method.

Sample	The SiO_2_ measured value of this method (%)		RSD (%)	National standard (%)	RE (%)
	Found	Average			
GBW07250	4.71 4.73 4.69 4.75 4.72 4.68 4.78	4.72	0.73	4.72 ± 0.07	0.06
GBW07251	8.37 8.41 8.36 8.44 8.33 8.35 8.42	8.38	0.49	8.35 ± 0.06	0.39
GBW07253	14.13 14.11 14.16 14.09 14.13 14.15 14.06	14.12	0.25	14.15 ± 0.07	−0.22
GBW07254	0.84 0.82 0.83 0.85 0.84 0.83 0.86	0.84	1.60	0.87 ± 0.02	−3.61
GBW(E) 070102	18.11 18.14 18.07 18.13 18.06 18.09 18.16	18.11	0.21	18.04 ± 0.08	0.38
GBW(E) 070106	7.63 7.65 7.69 7.73 7.72 7.71 7.66	7.68	0.50	7.68 ± 0.06	0.06

A method comparison experiment was conducted on actual samples (XS1, XS2, XS3, XS4, XS5) using both the proposed method and the standard method. Seven replicate measurements (n = 7) were performed. The relative standard deviation (RSD) of the silicon dioxide results was consistently below 3.04%. Furthermore, the results obtained by the two methods were in good agreement, with relative deviations below 2.71% (Table 4). These results comply with the requirements of the “Quality Management Specifications for Geochemical and Mineral Testing in Geological Laboratories”. For more data information, see S2 Table.

**Table 4:** The precision and accuracy of this method.

Sample	The SiO_2_ measured value of this method (%)		RSD %	National standard method %	Relative error %
	Found	Average			
XS1	4.13 4.13 4.09 4.12 4.11 4.08 4.14	4.11	0.54	4.12	0.14
XS2	5.16 5.10 5.08 5.09 5.14 5.09 5.11	5.11	0.58	5.11	0.00
XS3	6.67 6.61 6.58 6.63 6.57 6.56 6.62	6.61	0.59	6.62	0.22
XS4	0.54 0.56 0.58 0.59 0.56 0.55 0.57	0.56	3.04	0.58	2.71
XS5	14.59 14.65 14.52 14.57 14.63 14.58 14.59	14.59	0.29	14.67	0.55

A single-factor experimental design was employed to evaluate the effects of nitric acid and hydrofluoric acid volumes, water bath digestion time, boric acid dosage, and matrix matching on the performance of silica determination. Results showed that complete silica dissolution was achieved by digesting 0.2000 g of sample with 5 mL of nitric acid and 2 mL of hydrofluoric acid in a water bath for 30 minutes. Addition of 10 mL of 50 mg mL ⁻ ¹ boric acid effectively complexed excess fluoride ions, enabling accurate quantification by ICP OES. Method validation yielded a Limit of Detection (LOD) of 0.974 µg g ⁻ ¹ for silica. Analysis of five fluorite certified reference materials (GBW07250, GBW07251, GBW07253, GBW07254, GBW(E)070102, and GBW(E)070104) showed good agreement with their certified values. Furthermore, the results for five real fluorite samples were consistent with those obtained using the alkaline fusion spectrophotometric method.

## Conclusions

The method utilizes nitric acid–hydrofluoric acid digestion under water bath conditions for fluorite sample preparation, effectively minimizing silicon loss during digestion. The addition of boric acid complexes excess fluoride ions, preventing hydrofluoric acid from corroding the quartz components of analytical instruments. This allows the use of conventional ICP OES systems, which are otherwise incompatible with hydrofluoric acid-containing solutions. Based on these findings, a rapid method for the determination of silicon dioxide content in fluorite using ICP OES was developed. Method validation demonstrated that the measurement range for silicon dioxide was 0.0004% to 25%. The relative standard deviation (RSD, n = 7) obtained from actual sample analyses was ≤ 3.04%, and the relative deviation observed in comparative tests using different methods was ≤ 2.71%, both of which satisfy the requirements for practical analytical applications.

Compared with other methods for silica detection, the proposed method offers a simpler analytical procedure and lower cost, making it suitable for the rapid determination of silica in bulk fluorite samples. The application of boric acid to complex excess fluoride ions provides a viable approach for analyzing hydrogen fluoride medium samples using quartz-injection-based systems such as Inductively Coupled Plasma Atomic Emission Spectrometry (ICP OES) and Inductively Coupled Plasma Mass Spectrometry (ICP MS). However, a limitation of this method is its relatively low upper detection limit for silica. When silica content exceeds 25%, X-ray fluorescence analysis (XRF) or gravimetric methods are recommended for more accurate and reliable quantification.

## Supporting information

S1 Fig The impact of different volumes of nitric acid on the results of silicon dioxide. (XLSX)

S2 Fig The impact of different volumes of hydrofluoric acid on the results of silicon dioxide. (XLSX)

S3 Fig The impact of different digestion times on the results of silicon dioxide. (XLSX)

S4 Fig The impact of different volumes of boric acid on the results of fluoride ion. (XLSX)

S1 Table The RSD and RE of this method. (XLSX)

S2 Table The precision and accuracy of this method. (XLSX)
